# Development and Characterisation of Gastroretentive Solid Dosage Form Based on Melt Foaming

**DOI:** 10.1208/s12249-019-1500-2

**Published:** 2019-08-19

**Authors:** Gábor Vasvári, Ádám Haimhoffer, László Horváth, István Budai, György Trencsényi, Monika Béresová, Csaba Dobó-Nagy, Judit Váradi, Ildikó Bácskay, Zoltán Ujhelyi, Pálma Fehér, Dávid Sinka, Miklós Vecsernyés, Ferenc Fenyvesi

**Affiliations:** 1 Department of Pharmaceutical Technology, University of Debrecen, Nagyerdei krt. 98., Debrecen, H-4032 Hungary; 2 Department of Pharmaceutical Surveillance and Economics, University of Debrecen, Nagyerdei krt. 98., Debrecen, H-4032 Hungary; 3 Faculty of Engineering, University of Debrecen, Ótemető utca 2-4, Debrecen, H-4028 Hungary; 4 Department of Medical Imaging, University of Debrecen, Nagyerdei krt. 98., Debrecen, H-4032 Hungary; 5 Department of Oral Diagnostics, Semmelweis University, Szentkirályi út 47., Budapest, H-1088 Hungary

**Keywords:** solid foam, lipid matrix, PEG 4000, gastric retention, solid dispersion

## Abstract

Dosage forms with increased gastric residence time are promising tools to increase bioavailability of drugs with narrow absorption window. Low-density floating formulations could avoid gastric emptying; therefore, sustained drug release can be achieved. Our aim was to develop a new technology to produce low-density floating formulations by melt foaming. Excipients were selected carefully, with the criteria of low gastric irritation, melting range below 70°C and well-known use in oral drug formulations. PEG 4000, Labrasol and stearic acid type 50 were used to create metronidazole dispersion which was foamed by air on atmospheric pressure using in-house developed apparatus at 53°C. Stearic acid was necessary to improve the foamability of the molten dispersion. Additionally, it reduced matrix erosion, thus prolonging drug dissolution and preserving hardness of the moulded foam. Labrasol as a liquid solubiliser can be used to increase drug release rate and drug solubility. Based on the SEM images, metronidazole in the molten foam remained in crystalline form. MicroCT scans with the electron microscopic images revealed that the foam has a closed-cell structure, where spherical voids have smooth inner wall, they are randomly dispersed, while adjacent voids often interconnected with each other. Drug release from all compositions followed Korsmeyer-Peppas kinetic model. Erosion of the matrix was the main mechanism of the release of metronidazole. Texture analysis confirmed that stearic acid plays a key role in preserving the integrity of the matrix during dissolution in acidic buffer. The technology creates low density and solid matrix system with micronsized air-filled voids.

## INTRODUCTION

Several drugs display site-specific absorption in the gastrointestinal tract (1). Contribution of numerous factors, such as site specific, environmental pH and/or intestinal enzyme activity, could lead to low bioavailability (2). The gastrointestinal motility determines the residence time of the formulation in its absorption window (3).

Gastric retention is a promising mechanism for the oral modified-release drug products (4). These delivery systems provide sustained drug release coupled with resistance against the gastric milling (5,6) and emptying motions (7,8) and show prolonged residence time in the stomach. On the other hand, when localised therapy of the stomach or the duodenum is preferred, these delivery platforms may provide site-specific drug release for a longer time (9).

To achieve gastric retention, several technologies are available. Expanding devices increases their size upon or followed by the contact with gastric juice to inhibit transit through the pyloric sphincter. Hydrogels (5,10), and other unfolding technologies, such as the Accordion Pill® (7,11,12), are formulations with prolonged residence time in the stomach. Mucoadhesive formulations containing hydrophilic polymers adhere to the gastric mucosa (13) and release their drug in sustained manner.

Floating or low-density formulations remain on the top of the gastric content (14), thus avoiding passage to the duodenum. Gas-generating platforms usually contain carbonates (15–17) and polymers to entrap the formed gas or a balloon filled with volatile liquid. Nevertheless, the floating lag time, the time needed for the formulation to rise to the surface, might take a couple of minutes in some cases (18). Instantly floatable devices, on the other hand, have no lag time, they remain on the surface (9,19–21).

Up to date, promising new platforms and technologies to produce low density and sustained release formulations were developed. Hot-melt extrusion was successfully used to inflate extrudates with ethanol (9), carbonates (22,23) or pressurised carbon dioxide (24) . However, these processes were done at high temperature and the cutting or spheronisation was done after filament extrusion.

Our aim was to develop a simple and new technology, based on melt-foaming, which can be easily filled into the final dosage form, namely hard gel capsules. After filling, the foam quickly solidifies upon cooling and keeps its structure. In this work, a novel apparatus is presented and detailed for foaming a molten dispersion by mechanically dispersing gas into it. The main pharmaceutical attributes of the floating solid foams are also described.

## MATERIALS AND METHODS

### Materials

Polyethylene glycol 4000 (PEG4000), stearic acid, type 50 (SA) and metronidazole (MNZ) were Ph. Eur. grade and purchased from Molar Chemicals Ltd. (Halásztelek, Hungary). Labrasol® was kindly gifted from Gattefossé (Saint-Priest, France). Other reagents were all of analytical grade and purchased from Sigma Aldrich Kft. (Budapest, Hungary).

### Foaming Device Setup

**Fig. 1:**
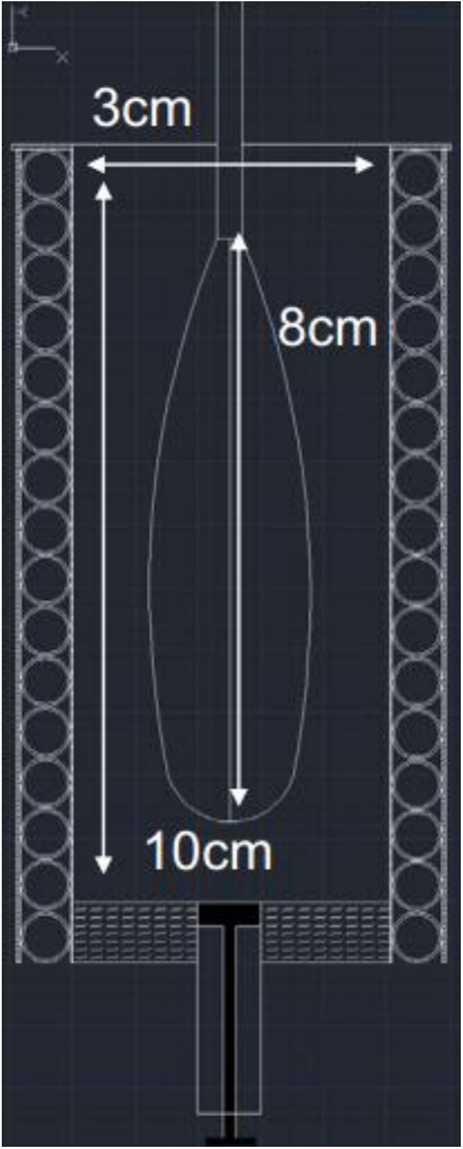
In-house apparatus with a whisker-type agitator used for melting, mixing, foaming and moulding the dispersion (3.0 × 10.0 diameter, height in centimetres, the difference between the shaft and the whisker head is 8.0 cm)

In-house apparatus was designed and built from polypropylene tube. The equipment is presented in Fig. 1. Briefly, the apparatus is a cylinder with the volume of 60 mL, which ends at the bottom in a 10-mm wide valve. The outer surface on the sides and at the bottom is water-jacketed with 6-mm plastic tubing. The jacket is connected to a Julabo F25 temperature control unit equipped with a Julabo ME circulator. The agitator is made of 1-mm wide stainless steel wires. The agitator is connected to an IKA EURO-ST D overhead stirrer. Dimensions and a schematic picture of the agitator are also presented in Fig. 1.

### Effect of Temperature on the Viscosity of the Molten Suspension

The apparent viscosity of the molten PEG4000 containing 30 m/m% MNZ was measured by a Rheolab QC rheometer (Anton Paar Hungary Ltd.) equipped with a concentric cylinder-jacketed measuring cell. The cell was connected to a Viscoterm VT 2 waterbath (Anton Paar Hungary Ltd.). The viscosity curves were recorded with the RheoPlus software. The measurement cell was heated to 65°C prior to loading with the molten and homogenous MNZ suspension. Viscosities were measured between 65 and 53°C with the difference of 2°C with constant shear rate of 1000 rpm. The maximum viscosity values were plotted as a function of temperature.

### Preparation of Solid Foams

Forty grams of the different compositions (M1-M7), presented in Table I, was foamed by the following method. PEG 4000, Labrasol® and SA were measured and loaded into the preheated foaming equipment with the slow agitation of 50 rpm at 65°C (jacket temperature). After complete melting, MNZ (average particle diameter, 180–125 μm) was dispersed for 10 min in the molten mixture, at 300 rpm. Cooling was started by setting the temperature of the water circulating in the jacket to 53°C. After 5 min, when the temperature of the water reached 53°C, foaming was done by heavy agitation at 2000 rpm and dispersing air into the molten mass. The procedure was done for 5 min maximum. For further investigations of the foamed matrix system, the foamed and hot dispersion was moulded into a steel mould (*V* = 1.027 mL, bullet shape) and cooled.

**Table I:** Compositions and Densities of the Foamed Compositions

Formulation	MNZ	SA	PEG 4000	Labrasol	Density (g/cm^3^) ± SD before foaming^*a*^	Density (g/cm^3^) ± SD after foaming^*a*^
M1	30%	0%	70%	0%	1.28 ± 0.0072	1.26 ± 0.0102
M2	30%	0%	68.5%	1.5%	1.27 ± 0.0073	1.14 ± 0.0195
M3	30%	0%	67.5%	2.5%	1.22 ± 0.0284	1.17 ± 0.0144
M4	30%	5%	63.5%	1.5%	1.22 ± 0.0178	0.89 ± 0.0341
M5	30%	10%	57.5%	2.5%	1.26 ± 0.0098	0.93 ± 0.0396
M6	30%	5%	65%	0%	1.27 ± 0.0093	0.82 ± 0.0261
M7	30%	10%	60%	0%	1.29 ± 0.0083	0.93 ± 0.0408

Prior to density determination tests, randomly selected samples from the solidified and moulded compositions were tested to confirm instant floatation in pH 1.2 acidic buffer. M1, M2 and M3 compositions failed on this preliminary test, and their densities were determined to be more than 1.00 g/cm^3^

^*a*^Data present average values and standard deviations (*n* = 15)

### Determination of the Densities of the Samples

The following method was used to determine the density of the moulded compositions, (unfoamed or foamed) based on the pycnometer method. Firstly, liquid paraffin was filled into a 100-mL volumetric flask, up to the graduation marking. The filling weight was precisely measured and the true density of liquid paraffin was calculated. The value of density was found to be 0.857 g/mL. The cavities of the steel mould were fully filled with pure PEG 4000. After scraping off the excess amount, the solid blocks were removed from the mould and their weights were recorded. Later, a sample was loaded into a 100-mL volumetric flask and appropriate amount liquid paraffin was added to fill the flask up to 100 mL. The volume of each sample was determined with the following formula:

$$ {V}_s=\frac{m_{p1}-{m}_{p2}}{\rho } $$

where *V*_*s*_ is the volume of the sample, *m*_p1_ is the weight of liquid paraffin which fills up the flask without an immersed sample, *m*_p2_ is the weight of liquid paraffin which fills up the flask with an immersed sample; *ρ* is the density of the liquid paraffin. Using the mass and volume data of the samples, their densities were calculated.

### SEM Analysis and Diameter Determination of Foam Cavities

Hitachi Tabletop microscope (TM3030 Plus) was used to characterise the solid foams (22). Samples were split in halves and were attached to a fixture with a double-sided adhesive tape containing graphite. Average diameter of the voids was calculated by measuring the diameter of hundred random cavities (GIMP 2.8 software) from at least 3 regions of the solid block.

### Microtomography

In order to characterise and visualise the internal microstructure of solidified molten foamed and unfoamed dispersion of M7 samples, the following protocol was developed. As described in ‘Preparation of Solid Foams’ section, the regular foaming process was done. A small portion of the homogenous dispersion was removed prior to the foaming step, from the jacketed vessel with a PET tube (internal diameter, 5.0 mm) attached to a 10-mL syringe, to obtain the unfoamed sample. After the foaming step, another portion was quickly and carefully removed from the vessel with a similar PET tube to prepare the foamed sample. The molten dispersions were allowed to cool down and solidify in the plastic tubes. After solidification, the rods of the solid samples were cut into 5–6-mm long cylinders. A random foamed and unfoamed cylinder was attached to each other with a soft glue and this preparation was scanned later. A SkyScan 1272 compact desktop microCT system was used for the measurement. Scanning parameters were the following: image pixel size, 5 μm; matrix size, 1344 × 2016 (rows × columns); source voltage = 50 kV; source current = 200 μA; flat field correction and geometrical correction were used. After scanning, SkyScan NRecon package (Version: 2.0.4.2) was used to reconstruct cross-section images from tomography projection images. Post-alignment, Beam-hardening correction, Ring artefact correction and Smoothing were done. The output formats were DICOM and BPM images.

### Dissolution Test

Nine hundred milliliters of hydrochloric acid media, pH: 1.2 without pepsin was selected for dissolution tests (16,25). Rotating paddle method with the rotation speed of 75 rpm and 37°C was set up in a dissolution tester (Erweka DT 800). Samples of 4 mL were withdrawn after 5 min, 15 min, 30 min, 1, 2, 3, 4, 5, 6, 7, 8 and 10 h. The samples were diluted with purified water and filtered through a 0.22-μm PES membrane syringe filter. The released amount of MNZ was determined by UV/VIS spectrophotometer (Shimadzu UV 1601, Shimadzu Corp. Kyoto, Japan) at 278 nm. Three random samples were selected for the tests from every composition. Floatation was inspected at the beginning, during and at the end of the test also.

### Water Uptake and Matrix Erosion Studies

Erosion and swelling properties of the solid formulations were determined by the following method. The initial weights of the samples (*W*_ini_) were recorded before the experiment, then they were placed into the dissolution vessels as described in the ‘Dissolution Test’ section. After 1, 3, 5, 7 and 10 h, samples were carefully removed with a plastic net and the weight of the wet samples (*W*_wet_) was measured after blotting the excess water. The samples were then dried in an oven (Memmert SFE 550, Memmert GmbH, Germany) at 45°C for 48 h, after cooling to room temperature, their constant weight (*W*_dry_) was measured (18). Three samples were tested from all compositions.

Water uptake % (WU %) was calculated by the following formula: (26)

$$ \%\boldsymbol{WU}=\frac{W_{\mathrm{wet}}-{W}_{\mathrm{dry}}}{W_{\mathrm{wet}}}\times 100 $$

Matrix erosion % and remaining masses of the foams were determined with the following formulas (27):

$$ {\displaystyle \begin{array}{c}\%\mathrm{Erosion}\frac{W_{\mathrm{ini}}-{W}_{\mathrm{dry}}}{W_{\mathrm{ini}}}\times 100\\ {}\%\mathrm{Remaining}=100\%-\mathrm{Erosion}\%\end{array}} $$

where *W*_wet_ means the weight of the wet samples, *W*_dry_ means the dried weight of the samples and *W*_ini_ means the initial weight of the samples, before test.

### Mathematical Analysis of the Drug Release Profiles

To compare the dissolution data of the different compositions, similarity or difference factors were calculated, as a model independent approach: similarity, f2 and difference, f1 factor was calculated for each composition. Dissolution efficacies were also calculated (28).

$$ \mathrm{f}1=\frac{\sum_{j=1}^n\mid {R}_j-{T}_j\mid }{\sum_{j=1}^n{R}_j}\times 100 $$

where *n* is the sampling number, *R*_*j*_ and *T*_*j*_ are the percent dissolved of the reference and the test products at each time point *j*.

$$ \mathrm{f}2=50\times \log \left\{{\left[1+\left(1/n\right){\sum}_{j=1}^n{w}_j{\left|{R}_j-{T}_j\right|}^2\right]}^{-0,5}\times 100\right\} $$

where *w*_*j*_ is an optional weight factor.

$$ \mathrm{DE}=\frac{\int_0^ty\times \mathrm{d}t}{y_{100}\times t}\times 100\% $$

where *y* is the drug percent dissolved at time *t*.

For the determination of release kinetics of MNZ, release data was fitted to zero-order, first-order and Korsmeyer-Peppas model equations.

$$ {\displaystyle \begin{array}{c}Q={Q}_0+{k}_0t\\ {}{Q}_t={Q}_0\times {e}^{-{k}_1t}\\ {}\frac{Q_t}{Q_{\infty }}={k}_{kp}{t}^n\end{array}} $$

where *Q* is amount of drug release at time *t*, *Q*_0_ is the initial amount of drug, *Q*_*t*_ is the amount of drug remaining at time *t*, and where *Q*_*t*_/*Q*_∞_ is fraction of drug released at time *t*. *k*_0_, *k*_1_ and *k*_kp_ are the kinetic constants for zero-order, first-order and Korsmeyer-Peppas models, respectively and *n* is the release exponent, indicative of the drug release mechanism. For Korsmeyer-Peppas model, only release data points were used in the analysis up to 60% drug release (29) .

### Statistical Analysis

For statistical analysis, GraphPad Prism® (Version 6.01, GraphPad Software Inc.) was used. Unpaired *t* tests were performed when two groups were compared, and one-way ANOVA was chosen when comparison of multiple groups was performed. Differences were considered significant at *p* < 0.05 (28).

### Dissolution Coupled Texture Analysis

Texture analysis was chosen to characterise the mechanical properties and structure of the dry and wetted foamed compositions. Dry samples were tested at 25°C without immersing them into acidic dissolution media. To monitor and determine hardness changes and erosion of the floating formulations, three random samples were placed onto dissolution vessels containing 900 mL of 37°C pH 1.2 hydrochloric acid media with 75-rpm paddle speed. To visualise water permeation into the matrix, the media was coloured with 20 drops of 5 m/m% Sicovit® Tartrazine (BASF) solution. The samples were carefully removed after 1, 3, 5, 7 and 10 h later and excess water was removed by using soft and plastic net and tissues. Wet and dry samples were analysed by the following method. Brookfield CT3 texture analyser was equipped with an acrylic cylinder, TA25/1000, (d: 50.8 mm) and the device was programmed to compress the blocks with constant speed (0.50 mm/s) until 4500 g of load. At the target pressure, the device fixed the probe for 5 s as a hold time. Following the hold time, the probe returned to its initial position; thus, the measurement took 20 s per sample. The load values were plotted in the function of time (s) to present the changes in the texture in real time.

## RESULTS

### Effect of Temperature on the Viscosity of the Molten Suspension

**Fig. 2:**
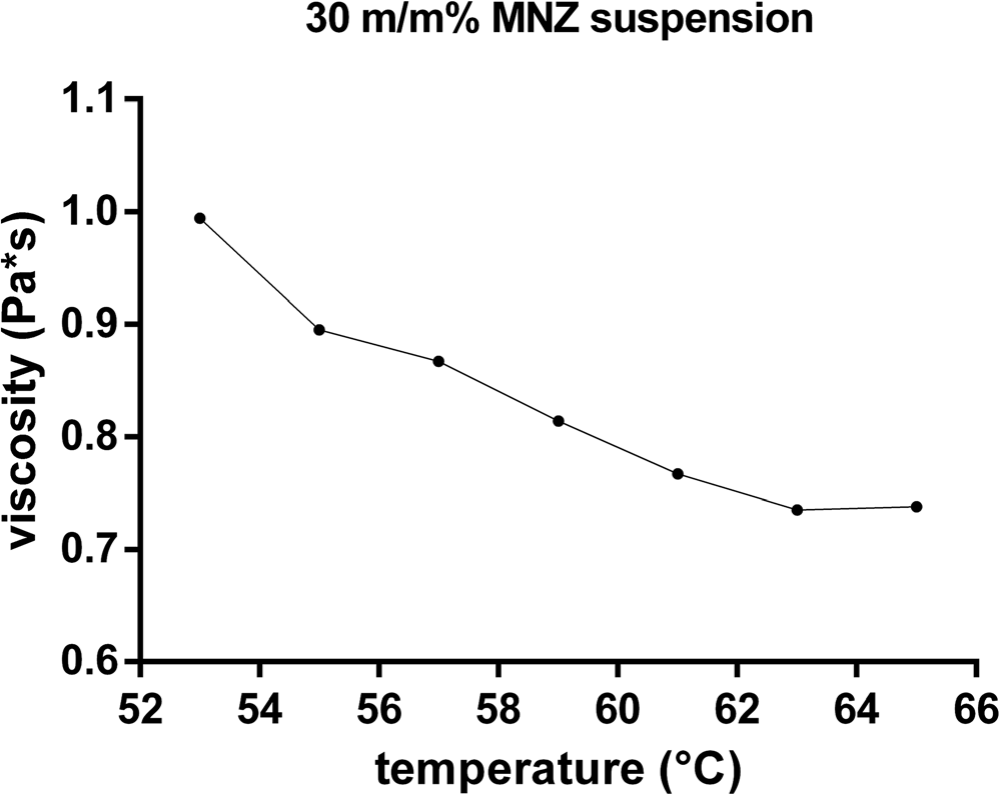
Viscosity temperature curve of the 30 m/m% MNZ- PEG 4000 suspension

Due to the polymeric nature of the PEG 4000 and to enhance the efficacy of gas entrapment in the molten dispersion, it was a key point to determine the viscosity-temperature relationship of the compositions. It is known that PEG 4000 has a melting point around 58–59°C (30,31). On the other hand, according to the specification of the European Pharmacopoeia, the freezing range is between 53 and 59°C. The effect of the MNZ on the viscosity changes of the solidification process was determined and the results are presented in Fig. 2. Fifty-three degree Celsius was found to show the highest viscosity values, namely 0.994 **(**Pa s**)**, which was suitable for mixing. It was also noticed that during the precise cooling, the viscosity values increased as the molten dispersion became semi-solid from its liquid state. As an optimal temperature to maximise gas entrapment efficacy, 53°C was chosen and later foam production was done at this temperature.

### Density Values of the Foamed Compositions

The density values and the average weights of the dispersions before and after foaming are also presented in Table I. It was found that M1, M2 and M3 are non-floating compositions, because their density results are higher than 1 g/mL. These compositions were excluded from further investigations. M4, M5, M6 and M7 were successfully foamed with this technology as their density values were below 1 g/mL and as a result, all of the foamed M4, M5, M6 and M7 compositions showed zero floating lag time in purified water. As a result of the foaming, the values of standard deviations increased as shown in Table I. The lowest density reached was 0.82 g/mL, this belongs to the M6 formulation. This means a 35.6% decrease in the mass, due to the dispersed gas. The average calculated API content in this formulation was found to be 252.3 mg.

### SEM Analysis and Void Characteristics

**Fig. 3:**
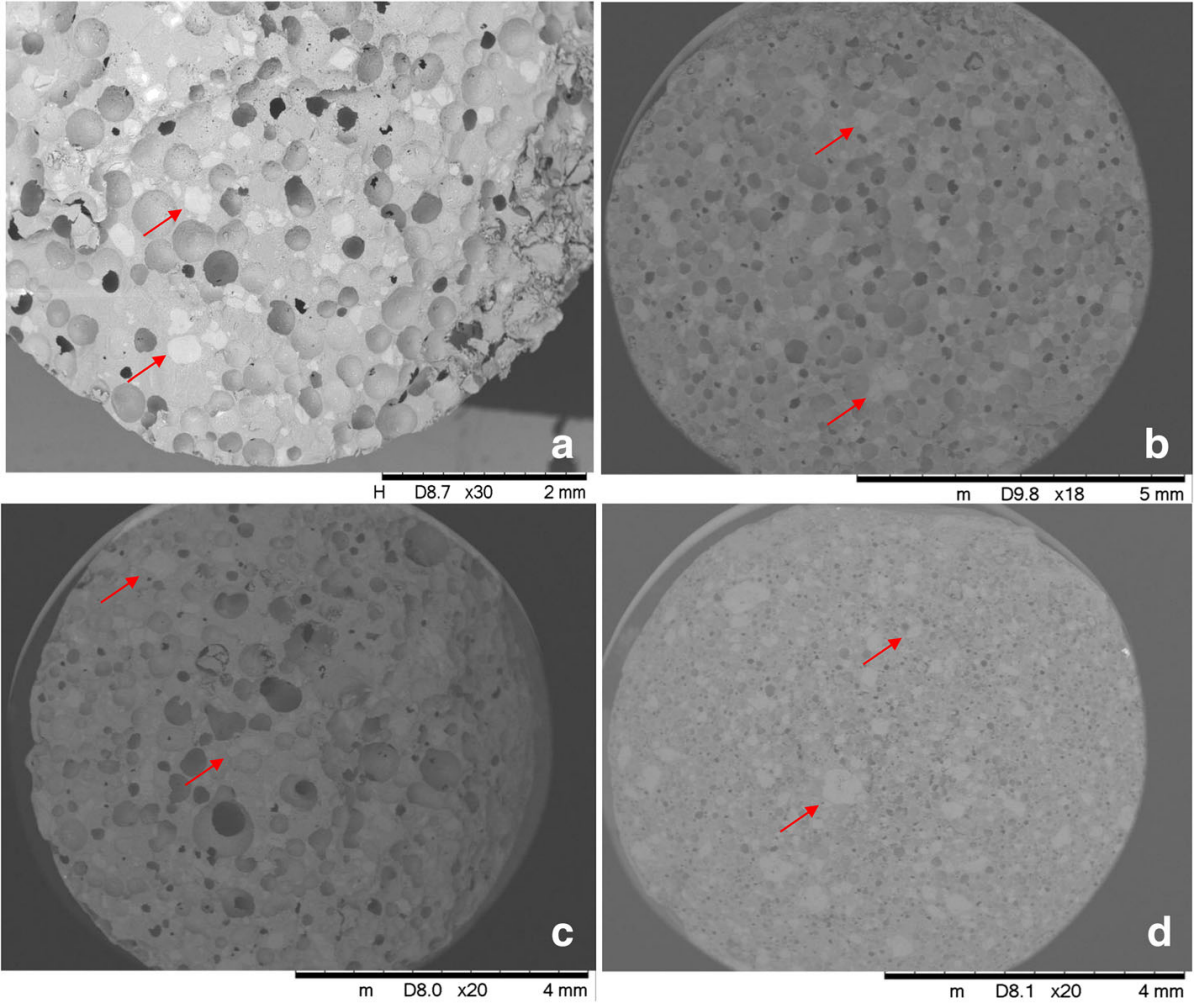
Representative SEM pictures of the M4 (**a**), M5 (**b**), M6 (**c**) and M7 (**d**) solid foams. Magnifications are between × 18 and × 30. Red arrows indicate the solid particles of MNZ in the solid foams

On the SEM images (Fig. 3), the cavities created in the melt by the dispersed gas phase can be easily distinguished from the matrix of the solidified melt and the crystals of the MNZ are also detectable. Owing to dispersing air into the molten material, the shape of the cavities is typically spherical or spheroidal. Cavities formed by the merging of bubbles may also be present. The cavities formed by merging of bubbles have short channel-like appearance, and they are assumed to originate from mechanically dispersing gas in the melt. The inner surfaces of the cavities are typically smooth, uneven surface can possibly be seen as well, as a result of the solidified but once fluid melt. The solidified melt forms one single phase in which the solid, undissolved crystals of the MNZ are present and the cavities created by the dispersed gas are distributed randomly. Particles of MNZ can be easily distinguished from the melted and solidified carrier and from the cavities created by the dispersed gas. It is not typical that any of the interfaces in the melt is enriched in solid particles. Scanning electron microscopy confirmed the development of a closed cell foam structure in the case of all foamed compositions. The structure of the foam is homogeneous and the smooth outer surface does not form a shell. The sizes of the voids were found to be 254 ± 83, 193 ± 63, 231 ± 113 and 67 ± 25 μm for M4; M5; M6 and M7, respectively.

### Microtomography Results

**Fig. 4:**
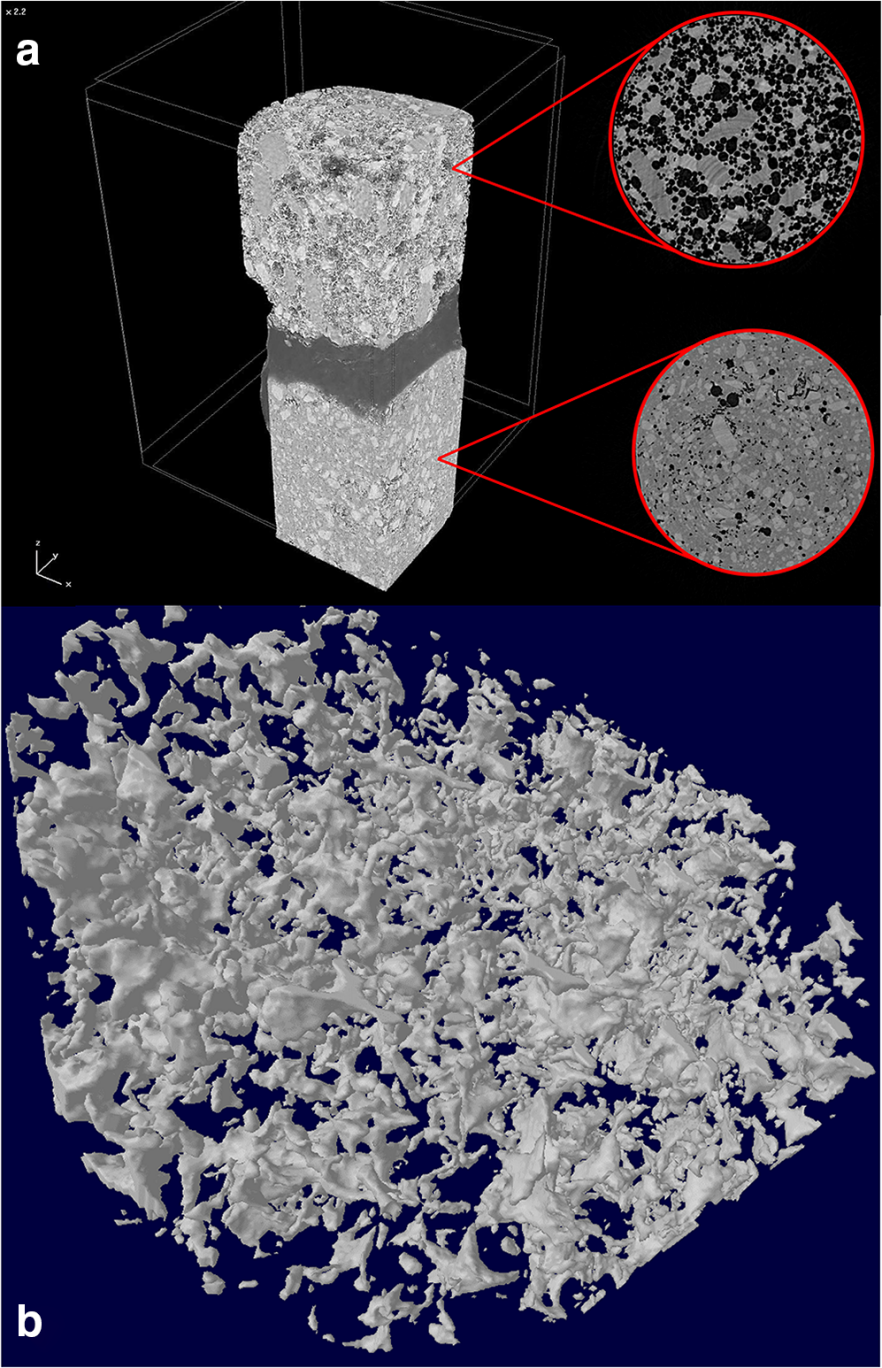
**a** Reconstructed microCT image of the M7 composition. The upper block represents the foamed product with a section from the original images, while the lower block represents the unfoamed product with a section from the original images. **b** Reconstructed model of the foamed melt presenting a closed-cell structure with interconnecting voids

Figure 4 shows the reconstructed image of the microCT scans performed on M7 composition. It is clearly seen that the process phase of MNZ dispersion incorporates tiny air bubbles into the molten dispersion which form voids in the molten matrix thereafter. On the other hand, the foaming process step creates a highly porous structure where the molten matrix is loaded with spherical or spheroidal bubbles. The distribution of the bubbles or voids is random. The reconstructed and computed model of the foam structure shows a closed cell structure in which interconnecting voids or deformed bubbles are present. These short channel-like voids could show various shapes, but most of them can be imagined as few chambers or rooms interconnected with tubular passages. However, none of them were found to be opened to the outer environment.

### Dissolution and Floating Properties

**Fig. 5:**
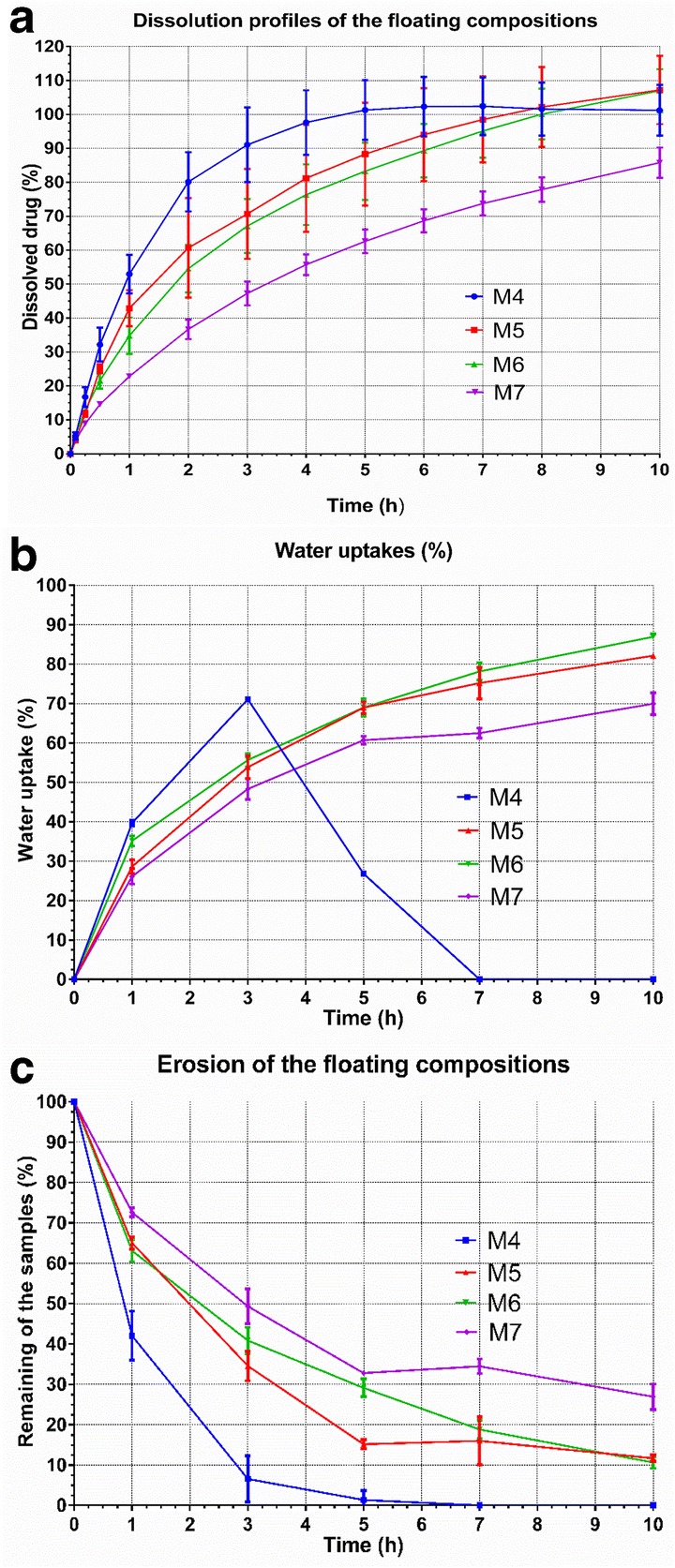
**a** Dissolution profiles of the floating foamed compositions. Bars represent mean ± S.D. (*n* = 3). **b** Water uptake (%) *vs.* time (h) curves of the floating foamed compositions. Bars represent mean ± S.D. (*n* = 3). **c** Erosion of the floating foamed compositions. Bars represent mean ± S.D. (*n* = 3)

During the dissolution tests, all samples were proven to possess zero floating lag time and none of them sank before complete disintegration. In the case of completely disintegrated M4 compositions, raft-like remnants were present at the top of the aqueous media. Dissolution properties (dissolution profile, results of the water uptake studies along with the erosion of the formulations) are presented in Fig. 5. M4 was found to release the MNZ in the shortest time, namely 91.07% were dissolved until 3 h. Water uptake curve of M4 greatly differs from the others. It was observed that the percentage of the water uptake of this formulation increased until 1 h, then a maximum uptake was reached at 3 h. At this time point, one of the samples completely eroded, and only two were removed and tested. The average of the percentage of remaining mass of the samples is only 6.56%. After 7 h, complete disintegration of the foamed matrix of M4 occurred. M5 released 88.33% of the drug after 5 h. Regarding its water uptakes and erosion, only 15.16% of the original mass of M5 was found in the dissolution media, with absorbing 68.97% water from the dissolution buffer. Water uptake maximums were found at 10 h, namely 82.16%, 86.95% and 69.94% for M5, M6 and M7, respectively. M6 released only 83.27% at 5 h, at that time point, 29.13% of the average initial weight was present in the dissolution vessel. Water uptake of the M6 samples continuously increased by time until the end of the test. M7 was found to release only 85.79% its MNZ content at 10 h. M7 adsorbed the least amount of water during this test and showed that the average of 26.92% mass remained after 10 h.

### Drug Release Analysis and Model Fitting

Dissolution efficiencies were calculated for each floating compositions and are presented along with the results of the pairwise procedure analysis in Table II. Comparison of the release profiles revealed that the drug release from M5 and M6 can be considered similar only (f1 and f2 are 4.92 and 67.87, respectively). When the dissolution efficiencies were calculated, it was found that for M4 showed the fastest release with the value of 88.43%, while for M7, the value of DE was only 57.21%.

**Table II:** Release Profile Comparison and Dissolution Efficiencies of the Floating Formulations

Dissolution efficiencies		Pair-wise comparison of dissolution profiles		
			f1^*a*^	f2^*b*^
		M4 *vs* M5	11.11	47.28
		M4 *vs* M6	15.48	41.06
M4	88.43%	M4 *vs* M7	36.88	25.84
M5	79.84%	M5 *vs* M6	4.92	67.87
M6	76.04%	M5 *vs* M7	28.99	33.92
M7	57.21%	M6 *vs* M7	25.32	38.02

^*a*^f1 values of the difference factor calculation

^*b*^f2 values of the similarity factor calculation

Drug release data were fitted to zero-order, first-order and Korsmeyer-Peppas models (Table III). Determination coefficients were used to determine the best fit. None of the models fitted to zero-order model, while the calculations revealed that the release data of all presented formulations fitted best to the Korsmeyer-Peppas model, since the correlation coefficients were all greater than 0.99.

**Table III:** Model Fitting Results of the Dissolution Data

Composition	Zero-order	First-order	Korsmeyer-Peppas
M4	0.7038	0.9875	*0.9932*
M5	0.7148	0.9479	*0.9967*
M6	0.7790	0.9836	*0.9989*
M7	0.8324	0.9947	*0.9985*

All values represent determination coefficients for each model fittings. Italic numbers indicate the highest values for each composition

### Texture Analysis

**Fig. 6:**
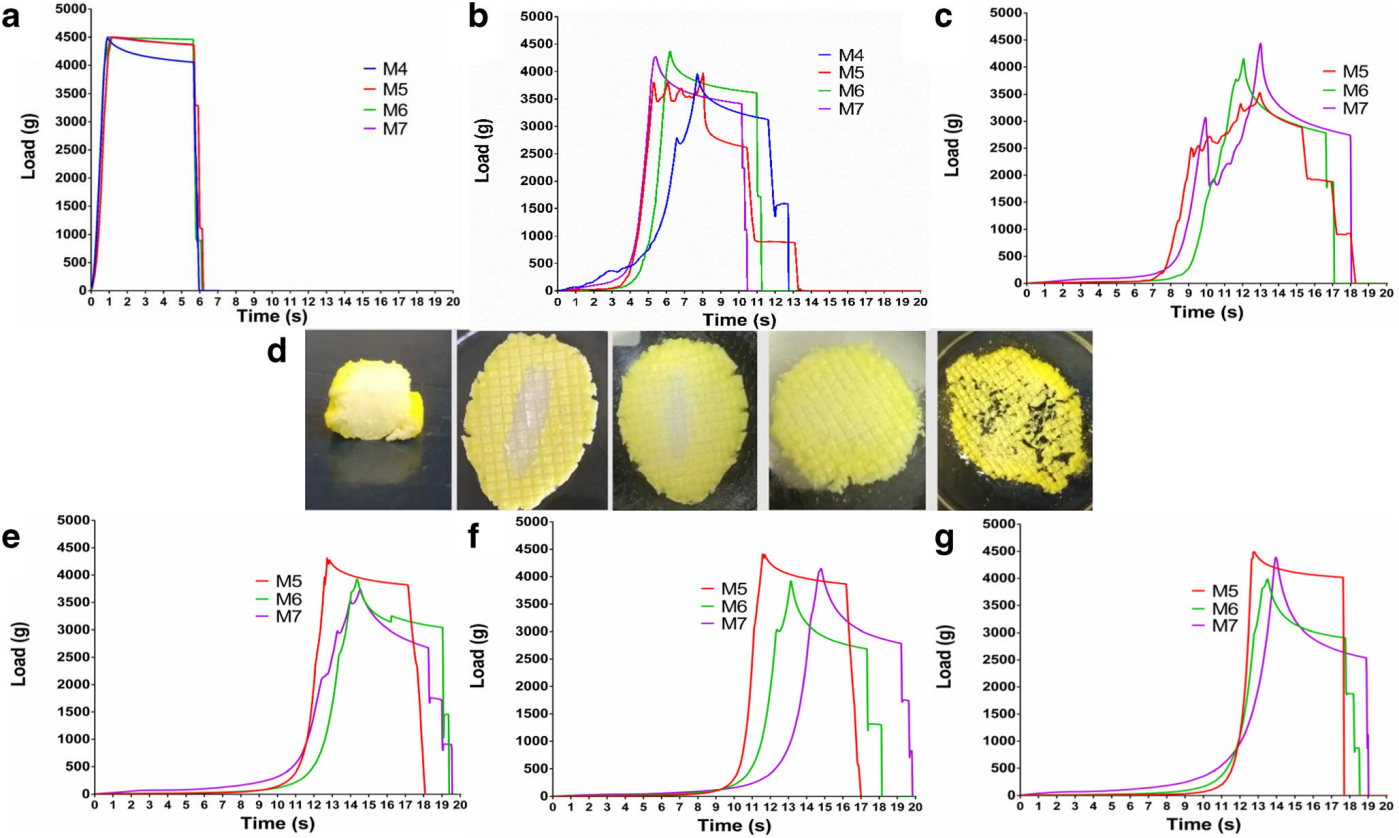
**a** Texture analysis result of the dry, foamed compositions at 25°C and dissolution coupled texture analysis result of the immersed samples at 37°C after 1 h (**b**), 3 h (**c**), 5 h (**e**), 7 h (**f**), 10 h (**g**). **d** Representative photos of M7 solid foams after compression by the measurement probe (from left to right: 1, 3, 5, 7 and 10 h), yellow stain was added into the dissolution media to follow the water permeation into the sample

Figure 6 represents the results of the texture analysis. It was revealed that in the cases of all floating compositions, a hard and resistant structure is present in spite of the air entrapment. Applying 4500 g of compression load on the foams did not result any cracks or fractures in the dry state at 25°C. On the other hand, when the compositions were compared, it was found that the deformation of M4 under the constant pressure of the analyser probe for 5 s is significantly different that the others, *p* < 0.0001. Regarding M4, a softer structure was found. As was expected on the basis of the erosion studies, M4 samples could be tested at only 1 h. M4 samples presented small dry and resistant cores inside, the compression of this core resulted a sharp drop in the load values, as seen on the diagram at 6.54 s (Fig. 6b). Those inner cores in the three parallel samples were small and fragile enough to be compressed and flattened by the moving probe. M5, M6 and M7 still contained dry, solid cores inside also, but their resistance against the compression force was different. When the M5 samples were tested, two of them contained dry cores resistant to the compression force, but one of them contained a fragile core inside which collapsed under the pressure of 3609 g and a series of smaller collapses took place until a more compacted structure developed to stop the measurement probe. M6 showed an outer pliable and deformable jacket-like outer part, compared with M7. M7 however presented a wetted outer layer which separated duo to the compressional test (Fig. 6d). After 3 h, all samples of M5, M6 and M7 were compressed and somewhat flattened by the measurement probe. M5 samples showed fragile inner cores which collapsed with a series of smaller collapses upon compression. The upslope of the curve starts at earlier time point, namely at 6.84 s indicating that the M5 compared with M6 (upslope starts at 8.92 s) and M7 has a softer structure. M7 at this time point also showed a more solid structure, since the load values are higher even at early time points, from 2 to 7 s. On the other hand, as represented with a photo on Fig. 6d, M7 lost a much of its integrity compared with the 1-h measurement. After 5 h, M7 contained only dry and brittle core, and from 2 to 11 s of the test time, higher load values were recorded. After 7 h, a nearly complete wetting and erosion of the samples resulted similar load-time curves for M5, M6 and M7. The only difference was found in the time coordinates of the turning points, namely 9.30, 10.18 and 12.20 for M5, M6 and M7, respectively.

## DISCUSSION

Our aim in this study was to design, build and use a batch type in-house apparatus to produce lipid-based monolithic matrices. We created low density, sustained release formulations with instant floating properties in acidic buffer without the need of gas generation and entrapment. After foaming, the formulation can be easily filled into the final dosage form, hard gel capsules, and then the foam quickly solidifies upon cooling and keeps its structure resulting the floating properties.

For the selection of the appropriate materials, we considered the following. The main matrix component must be solid at body temperature; therefore, it should possess mechanical resistance against the churning movements of the gastric contraction waves, because early matrix disintegration will lead to faster drug release (32). On the other hand, it should melt below 70°C, this temperature is a key factor. This temperature is the maximum filling temperature of hard gelatine capsules. At lower temperature, no risk of capsule shell damage (33) can occur and this attribute provides the possibility that the foamed dispersion can be filled into two-piece hard gel capsules. During our study, we applied a steel mould to study the foamed matrices and to discover its main pharmacological and physicochemical properties. The volume of each cavity in the mould (1.027 mL) and their dimensions are comparable with size 00 hard capsule (0.91 mL). Gastroretentive formulations must remain in the stomach to gradually release the API, which can be absorbed through the proximal part of the small intestine later (2,9). However, accumulation of the empty carriers should not happen; therefore, degradability or gradual disintegration (34) must be integrated into the matrix by careful design. PEGs, for instance PEG 4000, are a polymer with high degree of solubility in water and a melting range below 60°C. It is also favourable from technological aspect that it can exist in semi-solid state also, in which state, due to the high viscosity (35), dispersing gas into the fluid dispersion leads to a more efficient foam formation. Optimal temperature with sufficient ability to be moulded and to entrap gas was found for this polymer at 53°C.

In this study, we demonstrate that four different compositions with zero floating lag time were prepared with our apparatus in batch mode by filling the foamed and hot dispersions into metal mould. Labrasol for our experiments were selected as a non-ionic oil-in-water surfactant. In spite of its HLB value of 14, it has a decreased cytotoxicity compared with Polysorbates (36). It is well known as a solubiliser and o/w surfactant. It can be used for microemulsions and it is able to form light foams, as well (37). Based on our result, we can state that to sufficiently decrease the density of the dispersions, at least 5% SA was also elementary, 1.5% or 2.5 m/m% Labrasol alone could not decrease the densities below the density limit of 1.00 g/mL. However, we do not exclude the possibility that with increased agitation speed and shear force coupled with controlled gas injection, PEG-API gas dispersions can be created. Our apparatus disperse air at atmospheric pressure into the molten dispersion by cavitation and by vortex formation. During operation the wires of the agitator leaving and splashing into the swirling dispersion (38), this mechanism sucks and pulls air into the liquid in motion by breaking up the gas/liquid interface and also cause breakup of the previously dispersed bubbles. Addition of 5 m/m% SA to the PEG-MNZ dispersion decreased the density to the average value of 0.82 g/mL. This was a surprising and an unexpected result for us, which highlighted the importance of this material. The HLB value of the SA is 15 (39).

Besides its numerous application as a dissolution retardant (40) in oral hydrophobic matrix systems, SA when neutralised with tetrabutylammonium hydroxide can be used to create foam from o/w emulsions (41). The authors states based on the results of the experiments that SA contributes to the easier foamability due to two of its property. Firstly, SA, type 50 contains not only pure C18 fatty acid, this type of the excipient should contain at least 40.0–60.0% stearic acid, and the sum of the stearic acid and palmitic acid content must be at least 90.0%. The palmitic acid content results a lower melting temperature range, namely between 53 and 59°C compared with the pure SA, melting point, 69.6°C (39,42). This contributes to an increased viscosity at 53°C. Secondly, the presence of C18 and C16 fatty acids in the PEG matrix could also stabilise the air/PEG interface by accumulating at the surface of the air bubbles, thus creating an apolar layer which surrounds the dispersed gas phase. Similar mechanism has been earlier described and discussed when diary emulsion was foamed with a similar whipping technique (43,44). This hypothesis however should be confirmed by upcoming experiments.

The stabilization of the air/PEG interface can be detected when surface analysis was performed, adding SA alone results air bubbles in micron scale, while combining Labrasol with SA results an increase in the void sizes. On the other hand, open-cell structures were not created, but short and interconnecting clusters of spherical voids developed in all cases. None of the pictures showed that the dispersed crystals of MNZ were involved in bubble entrapment of interphase stabilisation, this is in concordance with the data of M1 composition presented in Table I. MicroCT scans on M7 foam structure confirmed a presence of a complex spongy structure. The foam structure however is not surrounded by a hard and thick, bubble-free shell-layer or outer jacket. Air was randomly distributed throughout the whole matrix. Signs of bubble coalescence, on the other hand, were detected. It can be stated on the basis of the results of imaging techniques, on Fig. 3 a, b and c, the interconnections are clearly seen on SEM pictures, while microCT scan of M7 confirmed the same structure with smaller voids (Fig. 4). SEM pictures also confirmed that MNZ remained in its crystalline form. Additionally, MNZ melts at 162.32°C (28), and no solid solution was prepared.

When the foamed compositions were investigated regarding their release characteristics, we found that the Labrasol increased the erosion and dissolution rates due to its good ability of micelle solubilisation (36). Regarding the water uptake studies, the PEG matrix absorbs water and swelling of the polymer chains develops; however, we should also mention that water can also penetrate and fill up the micronsized cavities. On the other hand, the whole inner pore system could not be completely flooded due to the shortness of the interconnections. This is favourable, since gas is entrapped in separate chambers providing continuous floatation. Dissolution of MNZ when compared with the matrix erosion curve suggests that drug release is mainly controlled by erosion. It can be stated on the basis of the presence of MNZ crystals and the low solubility of MNZ in water (45). On the one hand, it is noteworthy to mention that the diffusion path of the dissolved drug is elongated since air-filled voids are impermeable for the dissolution media, on the other hand, diffusion still contributes to the release of MNZ through the flooded channels and pores (1).

Comparison of dissolution data demonstrated that the release profiles of M5 and M6 can be considered similar only (f1: 4.92 and f2: 67.87) (46) in spite of their different composition and density (Table I), as well. Kinetic model analysis revealed that Korsmeyer-Peppas model fitted best to dissolution data (Table III): This kinetic model is used to analyse drug release of polymeric formulations when more than one type of release phenomena is involved. Release exponents of M4 and M5 were 0.9414 and 0.9759 indicate Super Case-II transport due to the cylindrical shape, while for M6 and M7, the *n* values were 0.7162 and 0.6889 corresponding to anomalous or non-Fickian diffusion transport. According to these findings, beside the rates of drug release, the mechanism is modified by Labrasol, as well. A possible explanation could be that Labrasol works as a plasticiser in the matrix; therefore, a rapid water uptake and matrix is coupled with the increased API concentration in the inner pores, due to the solubilisation of MNZ.

This also suggests differences in the speed of structural weakening of the foams as seen during texture analyses. Plasticising the PEG and SA chains together with a higher rate of water imbibition resulted the fastest erosion of M4. Plasticising effect could be the reason of the significantly softer texture of the dry M4 at 25°C. However, 10% of SA was able to develop a more ordered structure, resulting a harder and more resistant matrix. SA not only delays disintegration and increases hardness, but due to its edible nature, shows advantageous properties as oral release retardant (47).

## CONCLUSION

This study describes a novel technology developed to foam hot and molten dispersions on atmospheric pressure. This technology is directly applicable to produce floating, low-density moulded dosage forms. Undissolved drug in the molten dispersion does not affect the continuous buoyancy up to 30%. MNZ was released mainly by the erosion of the PEG-SA matrix; however, Labrasol as a non-ionic solubiliser alters the dissolution mechanism by increasing drug solubility and increasing the rate of water uptake. We applied several methods to characterise the properties of foam matrix system. SEM pictures and microCT scans confirmed that air bubbles form spherical closed-cell structure where clusters of interconnecting voids can be found. Texture analysis confirmed that SA in our matrices inhibits disintegration and maintains mechanical resistance in acidic buffer at 37°C. The temperature of the technology is ideal for hard capsule filling to prolong gastric residence time.
